# No increase in free fetal DNA level in ectopic pregnancy: A preliminary study

**DOI:** 10.4274/tjod.54715

**Published:** 2017-09-30

**Authors:** Özge Kömürcü Karuserci, Mete Gürol Uğur, Özcan Balat, Seyhun Sucu

**Affiliations:** 1 Gaziantep University Faculty of Medicine, Department of Obstetric and Gynecology, Gaziantep, Turkey

**Keywords:** Ectopic pregnancy, free fetal DNA, spectrophotometry

## Abstract

**Objective:**

The aim of this study was to diagnose ectopic pregnancy in the early period by measuring cell-free fetal DNA (cffDNA) levels in maternal blood using spectrophotometry.

**Materials and Methods:**

Thirty patients with ectopic pregnancy and 30 patients with first trimester intrauterine pregnancy were enrolled in this prospective controlled study. cffDNA levels in maternal serum were measured using spectrophotometry.

**Results:**

There were no differences between the two groups in terms of cffDNA absorbance levels.

**Conclusion:**

Spectrophotometry is not suitable for measuring cffDNA levels to diagnose ectopic pregnancies in the early period. Practical and cost-effective methods should be found or larger patient series should be investigated.

## PRECIS:

We have measured free fetal DNA level to diagnose ectopic pregnancy not to need repeat tests.

## INTRODUCTION

Ectopic pregnancy is complication of pregnancy with high morbidity and mortality rates, which is why early and precise diagnosis is very important. The current administration for diagnosis of ectopic pregnancy includes serial serum beta human chorionic gonadotropin (β-hCG) levels and transvaginal ultrasound^(1)^. However, it may not be possible to distinguish between intrauterine or extrauterine pregnancy in 8-31% of cases at the first examination^(2)^. Thus, several checks for β-hCG level and ultrasound monitoring are required for diagnosis and management decisions. Another marker could be used in diagnosis and management. To date, many researchers have worked on this subject and reported a range of new markers that could be used in this issue.

In recent years, cell-free nucleic acids have been studied for use as potential candidate biomarkers for numerous conditions, especially in gynecologic cancers, ovarian and endometrial diseases, obstetric disorders such as preeclampsia^(3)^, fetal aneuploidy, intrauterine fetal demise, and abortus^(4)^. Furthermore, concentration of cell-free nucleic acids in serum of patients undergoing in vitro fertilization (IVF) or embryo culture could give information about IVF outcomes^(5)^.

In this preliminary study, we aimed to measure and compare cell-free fetal DNA (cffDNA) expression in the maternal circulation among women with intrauterine and ectopic pregnancies using spectrophotometry for cheap, easy, early, and precise diagnosis with only one blood test.

## MATERIALS AND METHODS

This study was performed with 30 patients with ectopic pregnancy and 30 women with first trimester singular pregnancies without any medical problems serving as controls who were admitted to the Gaziantep University Faculty of Medicine, Department of Obstetric and Gynaecology. The ectopic pregnancy group was named as group 1 and the control group was named as group 2. An acceptance form was signed all volunteer pregnant women. This study was approved by the Gaziantep University Local Ethics Committee (approval number: 05/2011-16).

Diagnostic criteria of ectopic pregnancy were; irregular β-hCG increase, ectopic implantation of pregnancy or diffuse fluid in the pouch of Douglas and abdomen, as determined using transvaginal ultrasound.

We took 8-10 cc blood samples from the intrauterine pregnancy group and ectopic pregnancy group, which were then placed in two 15 cc Falcon tubes and sent to the laboratory within two hours and centrifuged at 2480 rpm for 10 minutes. The supernatant with its pellet was then separated to another Falcon tube and centrifuged at 3600 rpm for 20 minutes. Finally, the supernatant was separated into 3 Eppendorf tubes (1.5 cc) with its pellet and kept at -80 °C until required for analysis. These materials were thawed for analysis and DNA absorption measurements were made using a Tetra Spectrophotometer, Model: T80+UV/VIS Spectrophotometer.

### Statistical Analysis

Data were analyzed using SPSS 13.0. The independent t-test, independent Levene’s test, and Mann-Whitney U test were used when appropriate. Statistical significance was considered as p<0.05.

## RESULTS

**Figure 1:**
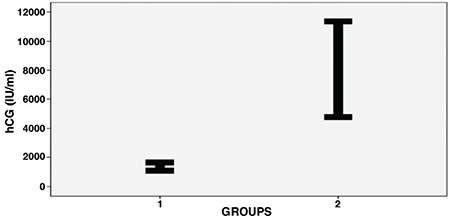
Beta human chorionic gonadotropin levels of two groups hCG: Human chorionic gonadotropin

**Figure 2:**
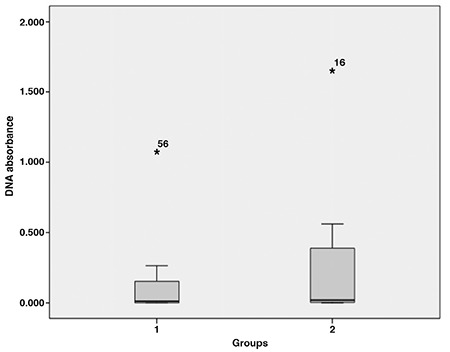
DNA absorbance levels of two groups *Values that are abnormally high but not statistically significant between the two groups

The average age of both groups was 24.9±4.8 years (range,16-36 years). The mean age of the ectopic pregnancy group was 25.3±4.5 years (range, 16-35 years). The mean age of the intrauterine pregnancy group was 24.4±5.1 years (range, 18-36 years). There was no statistically significant difference in terms of age between the two groups (p=0.776). The average weight of the both groups was 67.7±11.5 kg (range, 45-92 kg). There was no statistically significant difference in terms of weight (p=0.968), height (p=0.507), body mass index (p=0.873) and hemoglobin levels (p=0.741) between the two groups. The sociodemographic data of the groups are listed in Table 1 and Table 2. β-hCG levels were higher than in the control group and this was statistically significant (p<0.001) (Figure 1). There were no differences between the two groups with respect to DNA absorbance levels (p=0.647) (Figure 2).

## DISCUSSION

It is crucial to diagnose ectopic pregnancies in the earliest possible time because this interval of suspicion but uncertainity can cause dangerous conditions such as internal bleeding and future infertility^(6)^. Less than 50% of tubal ectopic pregnancies could be diagnosed at the first examination of patients^(7,8)^. We performed this research in the hope of identifying a new marker that could identify this important illness as early as possible. Therefore, we measured cffDNA absorbance using spectrophotometry, but we could found differenced between intrauterine the pregnancy and ectopic pregnancy groups in terms of cffDNA absorbance. We also found that the cffDNA values ​​did not increase with increasing hCG values. This is a valuable result because it showed that the amount of the cffDNA could not give information about the week or settlement of the pregnancy. A previous study also reported that cffDNA was not suitable as a marker for pregnancy complications, but it was suitable for the diagnosis of aneuploidies^(9)^.

Researchers are looking for many new markers to shorten the diagnosis time of ectopic pregnancy and reduce the possibility of tubal rupture. Previous studies researched placental (pregnancy-associated plasma protein A, human placental lactogen, inhibin A, activin A, vascular endothelial growth factor) and non-placental markers (glycodelin and vasculer endotelial growing factor) for the diagnosis of ectopic pregnancy^(10,11)^. Our study is the second regarding cffDNA in ectopic pregnancy. There is only one more study about cffDNA measurements in ectopic pregnancies. In that study, Lazar et al.^(12)^ investigated free DNA quantities using polymerase chain reaction (PCR) analysis of sex-determining region Y (SRY) between ectopic and intrauterine pregnancies. SRY was found in 15 ectopic pregnancies and 14 intrauterine pregnancies. Mean fetal free DNA quantity in ectopic pregnancies has been found higher than in intrauterine pregnancies (p<0.005). This result is significant but PCR is an expensive and long-term method; therefore, it is not suitable for ectopic pregnancy.

Measurement of the amount of nucleic acids is an essential tool in molecular biology that uses quantities of DNA solutions ranging from 1 pg/µL to 50 mg/mL^(13)^. A previous study measures the DNA quantity of Enterobacter from intestinal flora at 260 nm, but they used PCR at the beginning to extract genomic DNA^(14)^. We tried to measure cffDNA from maternal blood using spectrophotometry only. The reason that we found no meaningful result could be due to the fact that cffDNA in the blood of patients with ectopic pregnancy does not reach meaningful levels, different from intrauterine pregnancies, or that spectrophotometry only is insufficient without extracting cffDNA. Nevertheless, we foresee diagnosing ectopic pregnancy in the earliest period by measuring cffDNA with an easy, fast, and inexpensive method so we can use it in daily routine.

### Study Limitations

Spectrophotometry to measure cffDNA is a cheap method, but it may not be intensive enough or we need larger patient groups for searching.

## CONCLUSION

In conclusion, this pilot study may lead to other studies about cffDNA measurement for ectopic pregnancy in larger patient groups using spectrophotometry or any other easy, fast and cheap method.

The measurement of concentrations of cffDNA seems to be a promising tool for early diagnosis of ectopic pregnancy, but evaluation of the technique would be necessary. Spectrophotometry method is not suitable for measuring cffDNA levels to diagnose ectopic pregnancies in the early period. Practical and cost-effective methods should be found or larger patient series should be investigated for using cffDNA in the early diagnosis of ectopic pregnancy.

## Figures and Tables

**Table 1:**
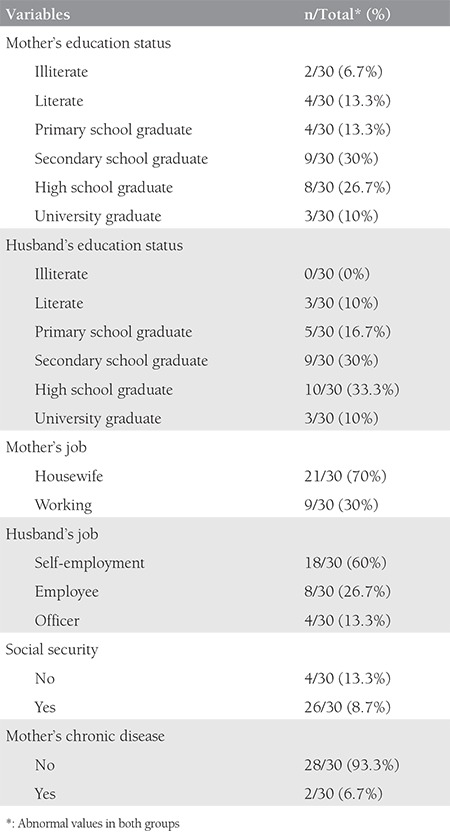
Sociodemographic data of ectopic pregnancy group (group 1)

**Table 2:**
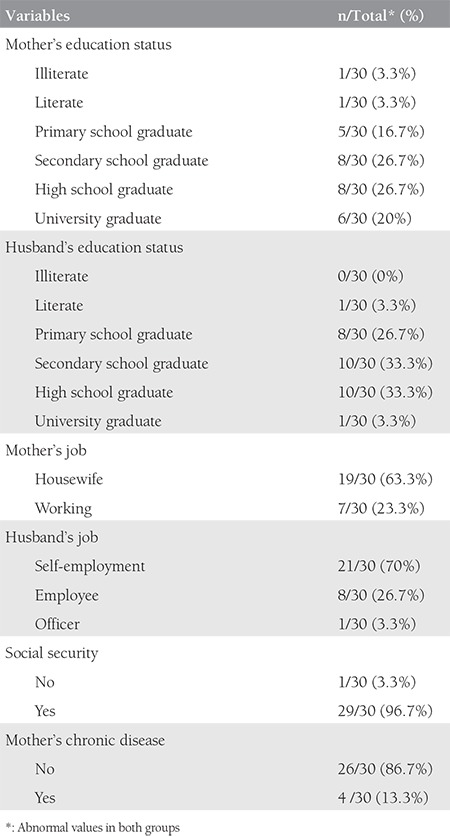
Sociodemographic data of intrauterine pregnancy group (group 2)
